# Bone Augmentation Procedures in a Patient with Acromegaly

**DOI:** 10.1155/2020/8479502

**Published:** 2020-07-01

**Authors:** Laura Andreea Schiller, Albert Cristian Schiller, Adalbert Schiller, Aida Gabriela Dascalu, Silviu Brad

**Affiliations:** ^1^Department of Radiology “Victor Babes”, University of Medicine and Pharmacy, Timisoara 300041, Romania; ^2^Department of Oral Surgery, Stomdas Clinic (Private Practice), Bacau 600125, Romania; ^3^Department of Prosthetic Dentistry “Victor Babes”, University of Medicine and Pharmacy, Timisoara 300041, Romania; ^4^Department of Endodontics, Stomdas Clinic (Private Practice), Bacau 600125, Romania; ^5^Department of Nephrology “Victor Babes”, University of Medicine and Pharmacy, Timisoara 300041, Romania; ^6^Department of Orthodontics “Victor Babes”, University of Medicine and Pharmacy, Timisoara 300041, Romania

## Abstract

The objective of this clinical case is to evaluate if bone augmentation procedures can be successful in a patient with altered bone metabolism due to a systemic disease: acromegaly. Two guided bone regeneration procedures were made on the same patient: horizontal ridge augmentation with lateral wall approach sinus graft of the left maxilla and horizontal ridge augmentation of the front left maxilla. CBCT assessment of the new formed bone was made after a minimum of nine months and dental implants were placed. The results show that bone augmentation procedures can be successful in a patient with acromegaly, after pituitary adenoma removal, when autologous bone is not included in the grafting procedure.

## 1. Introduction

Acromegaly is a syndrome caused by growth hormone excess in adults and is due to a growth hormone-secreting pituitary adenoma in the vast majority of cases [1].

The facial features of patients with acromegaly are characteristic: the nose is broadened and thickened, the malar bone becomes prominent, the lips are thick, and the facial lines are marked. Jaw malocclusion is a common phenomenon in these patients as there is a tendency towards mandibular overgrowth with prognathism, macroglossia, and teeth separation. Cortical bone thickens, and its porosity is decreased [2].

Studies show that acromegaly has a negative influence on trabecular bone, but not on cortical bone. Unfortunately, these studies are based on investigations made on skeletal bones, other than the maxilla and mandible: radius [3] and tibia [4]. A direct comparison of the maxillary and mandibular trabecular bone pattern factor with those of skeletal sites is not useful and would lead to false conclusions because of the difference in orientation of the sectional planes to the cancellous trabeculae [5].

Based on these facts, we could assume that acromegaly might have a similar negative influence on autologous bone grafts and less influence on other types of graft materials like anorganic bovine-derived bone mineral (ABBM) and allografts.

ABBM is a deproteinized, sterilized, bovine cancellous bone that is osteoconductive and provides a favorable scaffold for bone formation. It incorporates into the newly formed bone and, since it is slowly resorbed, keeps the volume of the graft very stable [6, 7].

A bone allograft is an osseous, transplanted tissue from the same species as the recipient but of different genotype [8]. Mineralized cancellous bone allograft (MCBA) is an effective osteoconductive material because its highly porous structure maintains volume [6, 7].

The purpose of this case report is to present the treatment and outcome of a patient with acromegaly and deficient maxillary bone that was rehabilitated using ABBM and MCBA.

## 2. Case Report

A 39-year-old patient addressed our clinic for complex oral rehabilitation with dental implants. He was early diagnosed with acromegaly due to a pituitary adenoma. The patient was treated by a medical team from the Netherlands that surgically removed the tumor and stabilized his condition. We began the surgical procedures after we had their approval.

**Figure 1:**
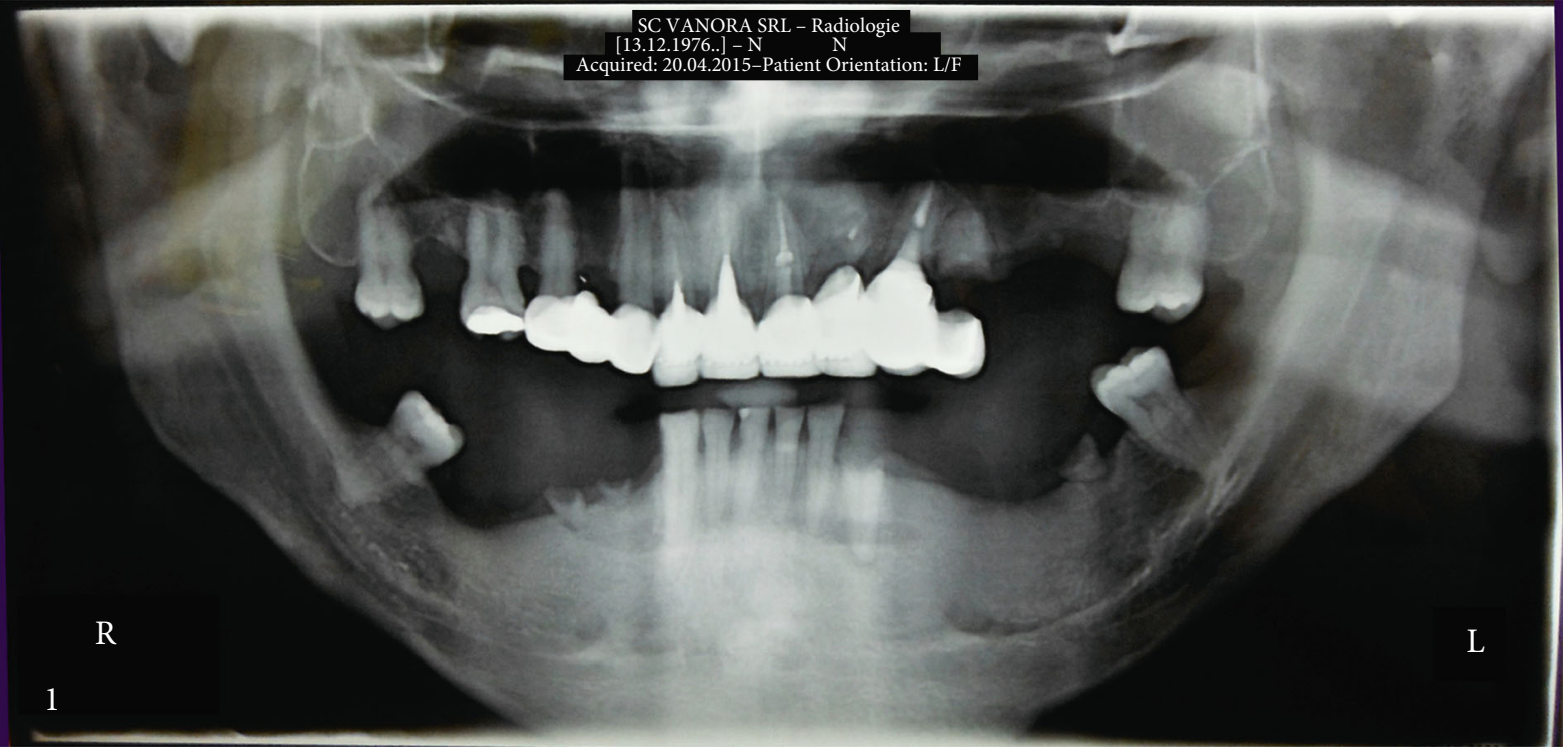
Initial orthopantomography.

The pretreatment we applied consisted of dental impressions, study models, orthopantomography, cone beam computed tomography (CBCT) of the left maxilla and of the left and right mandible, and laboratory investigations. The blood workup revealed 25-hydroxyvitamin D3 (25-OH vitamin D3) deficiency (15.64 *μ*g/L). We recommended vitamin D3 supplements (tablets 2000 U.I., 2/day, 60 days) until the values normalized. The initial image of orthopantomography is presented in Figure 1. The first stage of the treatment plan and workup was to reestablish oral health by removing old restorations, extracting unrestorable teeth (44, 45, 21, 22, 23, 24, 37, and 33) with addition of growth factors (Advanced Platelet-Rich Fibrin (A-PRF)), performing endodontic treatments, postcore restorations (15, 13, 12, and 11), and professional cleaning. The patient received provisional restorations: a fixed composite resin restoration with metal framework from tooth 15 to tooth 11 and a removable acrylic partial denture for the left maxilla.

**Figure 2:**
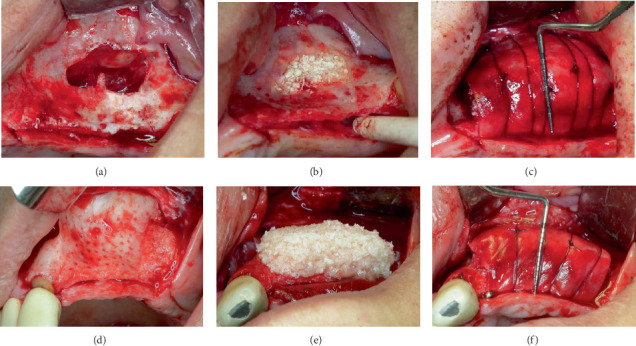
(a) Intraoral view of the elevated sinus membrane. (b) The sinus graft filling the space under the Schneiderian membrane. (c) Occlusal view of the horizontal ridge augmentation from the first surgery. (d) Labial view of the frontal defect, buccal perforations of the host bone, and, on the distal, the newly formed bone. (e) Occlusal view with the graft in place. (f) Occlusal view after stabilizing the graft with the resorbable membrane—second surgery.

The second stage of the treatment began 10 months later. The patient still suffered from 25-OH vitamin D3 deficiency (15.90 *μ*g/L) so we recommended to continue vitamin D supplements (tablets 2000 U.I., 2/day, 60 days) until the values normalized, followed by a smaller daily dose for maintenance (500 U.I.). We performed horizontal ridge augmentation with lateral wall approach sinus graft. After the incision and the full thickness flap, we created the lateral-access window of the sinus wall with a Piezotome, and we uncovered a small cyst (visible on the pre-op CBCT) and aspirated its content. There was a small perforation of the sinus membrane right next to the antral septa that closed spontaneously after elevating it, but we applied a resorbable collagen membrane for safety reasons. The host site was prepared for the graft with small holes through the buccal cortical plate (Figure 2), and then, we released the buccal flap with a periosteal incision. We introduced particulate bovine bone graft mixed with 150 mg/mL clindamycin solution (0.8 mL) and sterile saline into the space created for the sinus graft [8]. The horizontal component of the defect was augmented using a mix of 50% particulate bovine bone graft, 50% particulate freeze-dried bone allograft, A-PRF membranes, and A-PRF exudate. A second layer of particulate bovine bone graft mixed with sterile saline was placed over the previous one. We covered everything with a resorbable collagen membrane which we secured with titanium pins on the buccal and with resorbable sutures on the palatal side. We could not stabilize the pins next to the alveolar ridge due to the thinness of the bone. First, we sutured the apical buccal periosteum to the palatal flap with resorbable sutures for further stabilization of the graft, and then, the surgical site was closed with nonresorbable monofilament sutures. Broad spectrum antibiotics and nonsteroidal anti-inflammatory drugs (NSAIDs) were prescribed. The post-op healing was uneventful, and the sutures were removed at 14 days.

The third stage of treatment followed 8 months later. The patient started treatment with Sandostatin LAR prescribed by the endocrinologist. After evaluating the new bone formation on a CBCT, we saw that the first operation was a success and we moved to the next step: horizontal ridge augmentation of the front left maxilla. After the incision and the full thickness flap, we were able to see the newly formed bone. The host site was prepared for the graft with small holes through the buccal cortical plate, and then, we released the buccal flap with a periosteal incision. The horizontal component of the defect was augmented using a mix of 50% particulate bovine bone graft, 50% particulate freeze-dried bone allograft, A-PRF membranes, and A-PRF exudate (Figure 2). A second layer of particulate bovine bone graft mixed with sterile saline was placed over the previous one. We covered everything with a resorbable collagen membrane which we secured with titanium pins on the buccal and on the palatal side. First, we sutured the apical buccal periosteum to the palatal flap with resorbable sutures for further stabilization of the graft, and then, the surgical site was closed with nonresorbable monofilament sutures. Broad spectrum antibiotics and NSAIDs were prescribed. The post-op healing was uneventful, and the sutures were removed at 14 days.

**Figure 3:**
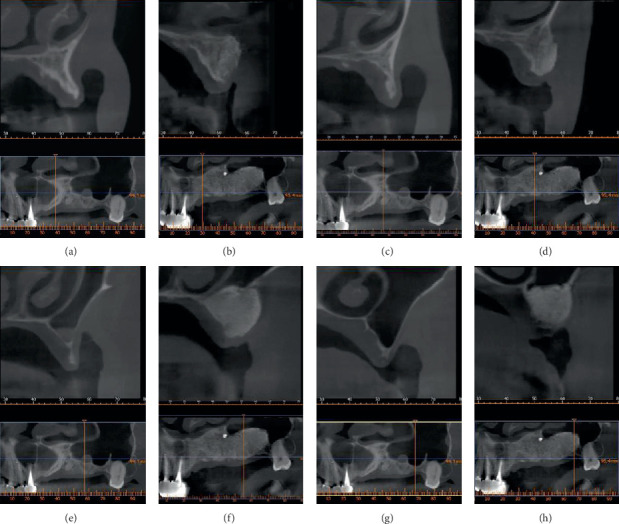
(a) Pre-op CBCT scan of the resorbed maxillary ridge on site 22. (b) Post-op CBCT scan of the newly formed bone on site 22. (c) Pre-op CBCT scan of the resorbed maxillary ridge on site 24. (d) Post-op CBCT scan of the newly formed bone on site 24. (e) Pre-op CBCT scan of the resorbed maxillary ridge on site 26. (f) Post-op CBCT scan of the newly formed bone on site 26. (g) Pre-op CBCT scan of the resorbed maxillary ridge on site 27. (h) Post-op CBCT scan of the newly formed bone on site 27.

The next stage of treatment started one year later, when the patient was reevaluated with a post-op CBCT which showed sufficient bone formation for implant placement (Figure 3). The blood workup revealed normal values for 25-OH vitamin D3 (29.91 *μ*g/L), and he was no longer on any medication. Unfortunately, there was a vertical bone loss due to the pressure from the removable provisional denture. Following the assessment of bone formation, we chose the dental implant sites and performed the implant placement: *22*: 3.5 × 11.5 mm, *24*: 4 × 13 mm, *26*: 4.5 × 13 mm, and *27*: 4.5 × 13 mm.

In Table 1, CBCT measurements show the amount of newly formed bone.

**Figure 4:**
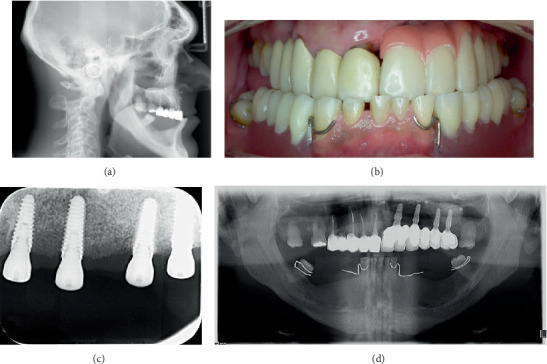
(a) Cephalometric radiograph made before implant placement. (b) Frontal intraoral view with the restorations. (c) Retroalveolar X-ray at implant uncovery. (d) Orthopantomography with the restorations.

The patient returned after one year, and the implants were uncovered. He received an implant-supported composite resin restoration with metal framework for the left maxilla and a removable acrylic partial denture for the mandible (Figure 4). All the above treatment steps and clinical decisions are in accordance with the appropriate EQUATOR guidelines (CARE checklist) and with the ethical standards of the responsible committee on human experimentation (institutional and national) and with the Helsinki Declaration of 1975, as revised in 2000, approved by the local ethics committee no. 1/28.05.2015.

## 3. Discussion

The best treatment option to rehabilitate the maxilla and correct the crossbite would have been an overdenture, but the patient refused. He also refused orthognathic surgery to correct the malocclusion; therefore, the treatment plan for the maxilla was a fixed dental prosthesis for the right side and a fixed implant-supported prosthesis for the left side. We did not consider zygomatic implants as an option, because the patient still had many restorable teeth on the right side and we consider it is unethical to remove restorable teeth to replace them with implants.

In order to rehabilitate the left maxilla with a fixed implant-supported prosthesis, surgical modifications were needed to change the highly deficient maxillary bone. The main concern of our complex intervention was the unpredictability of the bone augmentation outcome since the published data about bone augmentation procedures in patients with acromegaly are scarce. We discussed this with the patient and began with the most predictable procedure: horizontal ridge augmentation with lateral wall approach sinus graft.

Even though autogenous bone [9, 10] is considered to be the gold standard for bone grafts, we decided not to include it in the augmentation procedure because it was highly probable that the patient's bone structure was affected by intense remodeling due to his condition. We used anorganic bovine-derived bone mineral because is one of the most well-documented biomaterials and we mixed it with particulate freeze-dried bone allograft [11] to replace the autologous component. We took into consideration the fact that after a healing period of 9–10 months, the combination of deproteinized bovine bone mineral (DBBM) and a collagen membrane is an effective treatment option for horizontal bone augmentation before implant placement [12, 13].

The time between the stages of the treatment was longer than we would have wanted because the patient is not living in our country and came for therapy whenever possible. Furthermore, we made a removable provisional denture for the left maxilla at the patient's demand, only for aesthetic purposes. After each surgery, the denture was widely relieved and relined with soft material. The patient could not stay without denture for such a long time because of social reasons and that is why we decided not to reaugment the vertical discrepancy, considering that the result would have been the same. That is also the reason for which we did not use a different method. We were concerned that if we use a d-PTFE membrane [14, 15] with titanium reinforcement, bone block [16, 17] or bone shell [18, 19] techniques on a patient who is wearing a removable prosthesis over the grafted site, we would encounter graft exposure and infection. The fact that we could not see the patient on a regular basis because he lived in another country also influenced our decision-making. It might be possible that the bone formation would have been even better if the patient would not have worn a removable partial denture. It is a known fact that one of the most important conditions for a successful bone graft is stability, which is negatively influenced by the inherent mobility of the removable appliance.

We consider that the favorable outcome of our interventions is also related to the stable condition of the patient after pituitary adenoma removal.

Due to the complex nature of the case and the sagittal intermaxillary relations, we anticipated a difficult prosthetic rehabilitation, by maintaining the crossbite (Figure 4), because the patient refused orthognathic surgery and the overdenture treatment option. Upper and lower restorations must be prepared in crossbite occlusion in order for the masticatory forces to be transmitted to the jaw in an appropriate angle. In centric occlusion, posterior teeth make an 80-degree angle with the frontal and horizontal planes. If the angle is smaller than 70 degrees, crossbite is inevitable [20].

Another challenge that we had was with the quality of the radiographs and CBCT images: due to the atypical anatomy of the patient, the alignment in the X-ray machine was less than ideal. That is why we had to do multiple exposures, so we could analyze the images to the best of our ability.

The initial treatment plan included vertical and horizontal bone augmentation procedures of the right and left mandible and fixed implant-supported restorations after bone formation, also replacing the provisional fixed dental prosthesis from the right maxilla with a definitive metal-ceramic one. The patient could not follow through with this option due to financial reasons; therefore, the removable acrylic partial denture and the fixed composite resin restoration with metal framework we made as provisional prostheses became definitive ones. We first made an implant-supported metal-ceramic restoration for the left maxilla, but ended up changing it to a composite resin with metal framework restoration due to the overall weight of the superstructure. Unfortunately, all of these changes lead to less than desirable aesthetics.

## 4. Conclusion

In this case report, we were able to show that guided bone regeneration procedures like horizontal ridge augmentation with lateral wall approach sinus graft and horizontal ridge augmentation of the front maxilla can be successful in a patient with acromegaly when autologous bone is not included in the grafting procedure and the patient has a stable condition after pituitary adenoma removal. Unfortunately, the follow-up period of the augmented bone is not very long (two and a half years for the first surgery) and we do not have any follow-up after the delivery of the restoration.

For now, we have a reason to say that in the presented report, bone regeneration procedures were successful, but we cannot make any statement about implant survival in the augmented bone. More research is needed in order to improve the predictability of this type of interventions.

## Figures and Tables

**Table 1:** Evaluation of bone width on the CBCT.

Implant site	Before augmentation procedure (mm)	After augmentation procedure (mm)
22	1.6	6.0
24	2.2	7.0
26	1.4	7.9
27	2.3	8.6

## Data Availability

The authors confirm that the data supporting the findings of this study are available within the article and its supplementary materials.
